# Lithium‐induced kidney injury and fibrosis: A versatile model to explore cellular pathways of injury and repair

**DOI:** 10.14814/phy2.70552

**Published:** 2025-09-10

**Authors:** Paulomi Mehta, Tania Slatter, Robert Walker

**Affiliations:** ^1^ Kidney Injury and Transplant Immunology Group, Centre for Transplant and Renal Research Westmead Institute for Medical Research Westmead New South Wales Australia; ^2^ Department of Medical Laboratory Science, School of Pharmacy University of Otago Dunedin New Zealand; ^3^ Department of Medicine, Dunedin School of Medicine University of Otago Dunedin New Zealand

**Keywords:** amiloride, cell cycle arrest, cell proliferation, glycogen synthase kinase 3β, interstitial fibrosis, lithium

## Abstract

Lithium‐induced kidney injury is commonly associated with the development of nephrogenic diabetes insipidus. Longer term lithium exposure is associated with the development of chronic interstitial fibrosis. The mechanisms of lithium‐induced kidney injury are multifaceted, affecting many intracellular cell signaling pathways associated with cell cycle regulation, cell proliferation, and subsequent increased extracellular matrix formation and interstitial fibrosis. Amiloride has been demonstrated to have multiple pleiotropic actions, independent of its competitive antagonism of ENaC, in reducing the progressive lithium‐induced interstitial fibrosis. Further exploration of these interactions has the potential to expand our knowledge of pathways of tubular cell injury and repair, which in turn will lead to potential new therapeutic targets and drugs.

## 1 INTRODUCTION

Lithium salts (carbonate or chloride) have been used since the late 1940s and 1950s to effectively treat manic episodes associated with bipolar disorders (Cade, 1949; Schou et al., 1954) and is used as a first‐line prophylactic treatment to stabilize depression associated with BD (Berghöfer et al., 2013; Lewitzka et al., 2015; National Collaborating Centre for Mental Health, 2006). Despite the therapeutic effectiveness in controlling the underlying mood disorder, most patients (70%–90%) report the common side effects of nausea, tremor, and weight gain. About 19% of patients on lithium develop subclinical hypothyroidism due to lithium‐induced interference with cellular processes involved in the release of thyroid hormone (Shine et al., 2015). Up to 60% of individuals on lithium treatment report impaired urinary concentrating ability, which is evident within 1–2 years of commencing therapy. Of these, a number will develop overt nephrogenic diabetes insipidus (NDI) (Thompson et al., 1997; Walker et al., 2005) with about 40% of patients subsequently developing irreversible NDI (Thompson et al., 1997), even if lithium therapy is stopped.

A smaller percentage will develop a slowly progressive chronic interstitial fibrosis. Kidney registry data from around the world suggest that long‐term lithium exposure is responsible for about 0.8% of individuals with end‐stage kidney disease (Bendz et al., 2010). While higher therapeutic concentrations are associated with a greater probability of developing chronic kidney disease (CKD), it can occur with any long‐term exposure to lithium. Changes in estimated glomerular filtration rate (eGFR) are a relatively late manifestation and are only evident after 10–15 years of lithium therapy (Tondo et al., 2017). Once eGFR is below 45–50 mL/min/1.73m^2^, stopping lithium therapy does not modify the ongoing slow rate of progression of CKD (Markowitz et al., 2000; Presne et al., 2003). Therefore, if lithium therapy is efficacious in controlling the mood disorder, stopping lithium therapy needs to be discussed with the patient, who may well consider the benefits of having the mood disorder well controlled far outweigh the low risk of progressive CKD.

## 2 LITHIUM AND NEPHROGENIC DIABETES INSIPIDUS

In normal healthy individuals, the final urine volume is closely regulated at the level of the principal cell of the collecting tubule by the actions of arginine vasopressin (AVP). The collecting duct is usually impermeable to water. AVP binding to its receptor, V2R, activates adenylyl cyclase with the formation of cAMP, which in turn activates protein kinase A with subsequent phosphorylation of AQP2, enabling AQP2 trafficking and insertion to the apical membrane. Insertion of AQP2 on the apical side allows water to move down a concentration gradient, exiting the principal cells via AQP3 and AQP4, which are constitutively expressed on the basolateral membrane of the principal cells, to re‐enter the circulation (Boone & Been, 2008).

Lithium enters the principal cells via ENaC and inhibits the translocation of intracellular AQP2 to the apical membrane as well as longer‐term downregulation of AQP2 mRNA and synthesis (Christensen et al., 2011). Lithium inhibits vasopressin‐sensitive adenylyl cyclase (Christensen et al., 2011; Marples et al., 1995), possibly via competition for Mg2+ or via lithium‐induced inactivation (phosphorylation) of glycogen synthase kinase 3β (pGSK3β) (Marples et al., 1995), which in turn prevents the phosphorylation and activation of protein kinase A and the subsequent translocation of AQP2 to the apical membrane (Marples et al., 1995; Rao et al., 2010). Longer term, there is decreased AQP2 RNA expression, probably via further inhibition of adenylyl cyclase (AC) (Kaiser & Edemir, 2020; Rao et al., 2010).

## 3 AMILORIDE IN MODIFYING LITHIUM‐INDUCED NEPHROGENIC DIABETES INSIPIDUS

In 1985, Batlle et al. first demonstrated that lithium‐induced NDI could be modified using amiloride in nine patients (Batlle et al., 1985) but the mode of action was not clear. Since then, our understanding of collecting duct physiology has developed substantially with the identification of epithelial sodium channels (de Varez la et al., 2000) and aquaporins (Brown, 2003). Amiloride has been shown to act as a competitive inhibitor of ENaC (Vidt, 1981). In a small randomized controlled trial, Bedford, Weggery, et al. (2008) demonstrated that amiloride significantly reversed lithium‐induced NDI in 11 patients receiving lithium treatment. This reversal was associated with an increase in urinary excretion of AQP2, a variable decrease in urinary cAMP concentration that did not correlate with urinary AQP2 concentrations, and resetting of the medullary osmotic gradient. They inferred that amiloride, binding to ENaC, blocking the uptake of lithium into the principal cells, downregulated the lithium‐induced inhibition of cAMP generation by AVP, allowing more AQP2 to be transported to the apical cell surface and the subsequent improvement in kidney concentrating ability (Bedford, Weggery, et al., 2008).

**FIGURE 1:**
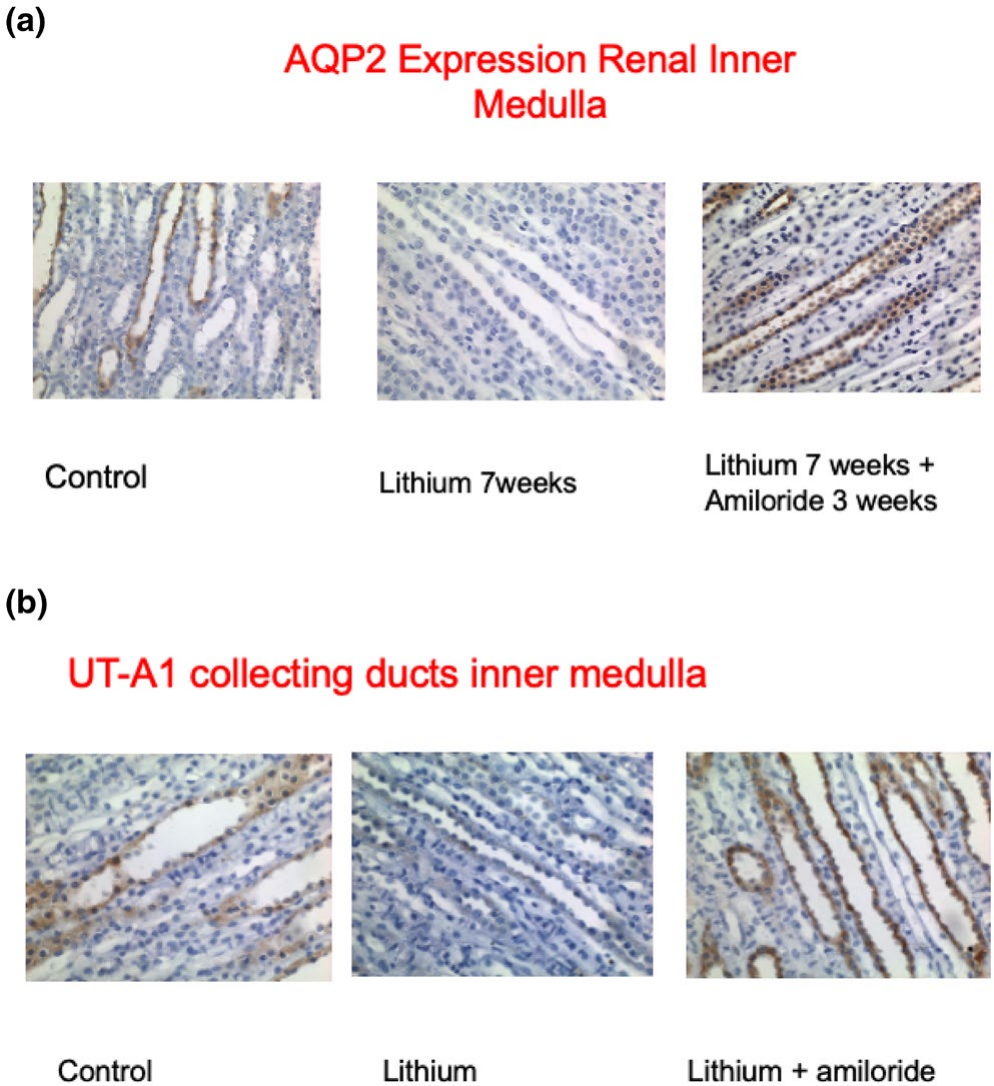
(a) Immunohistochemical detection of AQP2. (b) Immunohistochemical detection of urea transporter A1 (UT‐A1) in the renal inner medulla of control rats, rats fed lithium carbonate (60 mmol/kg dry food) alone for 7 weeks or lithium carbonate (60 mmol/kg drf food) for 7 weeks with amiloride (0.2 mmol/L) added to drinking water for the last 3 weeks. (magnification ×200) Modified from Bedford, Leader, et al. (2008).

This was further supported, experimentally in a rat model (Bedford, Leader, et al., 2008), where following 4 weeks of lithium exposure, there was a substantial reduction in the concentration of renal medullary organic osmolytes (urea, inositol, sorbitol, GPC, and betaine) (Table 1). This was associated with reduced expression of aquaporins AQP2, AQP3, and urea transporters (UT‐A1). This demonstrated that the inability of the animals to concentrate urine was not only due to a loss of the ability to modulate the water permeability of the principal cell apical membrane but was also due to the severe reduction of the medullary osmotic gradient. In the same series of experiments, treatment with amiloride resulted in a substantial restoration of the urinary concentrating ability, due to upregulation of AQP2 expression and enhanced expression of UT‐A1 in the medullary collecting ducts and the amounts of medullary intracellular organic osmolytes, despite the ongoing administration of lithium (Bedford, Leader, et al., 2008) (Figure 1).

**TABLE 1:** Osmolytes in renal medulla of rats with lithium‐induced NDI and rats with lithium‐induced NDI given drinking water containing 0.2 mmol/L amiloride.

	Control	60 mmol lithium	60 mmol lithium + 0.2 mmol/L amiloride
Inositol (mmol/kg^−1^ protein)	221 ± 35	85 ± 10*	179 ± 8^§^
Sorbitol (mmol/kg^−1^ protein)	35 ± 9	3 ± 1*	7 ± 2
GPC (mmol/kg^−1^ protein)	352 ± 80	91 ± 20*	231 ± 38^§^
Betaine (mmol/kg^−1^ protein)	69 ± 11		84 ± 10
Urea (mmol/kg^−1^ protein)	2868 ± 624	480 ± 117*	2132 ± 184^§^
Urine Osmol (mosm/kg)	1121 ± 90	287 ± 19*	1132 ± 154

*Note*: Rats fed lithium (60 mmol/kg dry food) alone for 7 weeks compared to rats fed lithium (60 mmol/kg dry food) for 7 weeks and amiloride 0.2 mmol/L (drinking water) for the last 3 weeks. Values mean ± SE. Modified from Bedford, Leader, et al. (2008).

* *p* < 0.05 compared to control.

^§^ *p* < 0.05 compared to lithium alone.

## 4 LITHIUM AND INTERSTITIAL FIBROSIS

Long‐term lithium therapy is associated with the development of chronic interstitial fibrosis and focal glomerulosclerosis (Aurell et al., 1981; Hestbech et al., 1977; Presne et al., 2003; Walker et al., 2013). These features of CKD are irreversible and not modified by the cessation of lithium therapy (Presne et al., 2003; Tondo et al., 2017). These features have been replicated in long‐term animal studies. Studies from our research group have demonstrated progressive proteinuria with lithium exposure and subsequent focal segmental glomerulosclerosis evident following 6 months exposure to lithium, consistent with lithium‐induced podocyte injury (Kalita‐De Croft et al., 2018; Walker et al., 2013). Although GSK3β is essential for podocyte integrity and lithium is known to partially inhibit GSK3β activity by phosphorylation, Hurcombe and colleagues have shown that this is not the main mediator of podocyte injury (Hurcombe et al., 2019). Clearly, further studies are required to sort out the downstream pathways that are modified by lithium‐induced GSK2β inhibition that modulate podocyte function (Hurcombe et al., 2019).

A number of studies have demonstrated that upon kidney injury, inflammatory and fibrotic processes are activated, promoting adaptive repair processes, including cell proliferation and tissue remodeling that usually leads to appropriate resolution of the injury. However, persistent injury can result in a maladaptive response, where kidney tubular epithelial cells undergo cell cycle arrest in the G2/M phase (interphase to mitosis) (Yang et al., 2010) leading to cell damage and persistent upregulation of pro‐inflammatory cytokines such as TGFβ and CTGF, leading to chronic fibrosis with excess extracellular matrix deposition (Yang et al., 2010).

Likewise, studies have demonstrated that lithium induces cell cycle growth arrest in the G2/M phase. Earlier studies by Christensen and colleagues reported changes in cellular composition of the collecting duct cells with increased proliferation of principal cells and dedifferentiation into intercalated cells (Christensen et al., 2004, 2006). Kjaesgaard and colleagues demonstrated that the epithelia lining the atypical tubular microcysts seen in human kidney biopsies from individuals on long term lithium therapy stained positive for proliferating cell nuclear antigen (PCNA) and pGSK3β suggesting GSK3β inhibition was associated with this altered cell proliferation (Kjaersgaard et al., 2012). de Groot and colleagues, using collecting duct cell cultures, demonstrated that 3 days of lithium exposure (1 mM lithium chloride basolateral aspect, 10 mM apical aspect) resulted in a significant increase in PCNA abundance and an increased abundance of phospho‐Histone H3 (p‐Histone 3) expression, consistent with these proliferating cells entering G2/M growth arrest (de Groot et al., 2014). The same authors demonstrated similar findings in vivo using a mouse model with lithium treatment for 13 days, inducing G2 growth arrest with approximately 40% of principal cells positive for p‐Histone 3 expression (de Groot et al., 2014). This was associated with increased Wnt‐β catenin (related to inhibition of GSK3β) activity and upregulation of cyclins associated with cell cycle regulation (de Groot et al., 2014). Collectively, these studies support the idea that the maladaptive cell cycle arrest is a postulated mechanistic pathway leading to kidney fibrosis.

**FIGURE 2:**
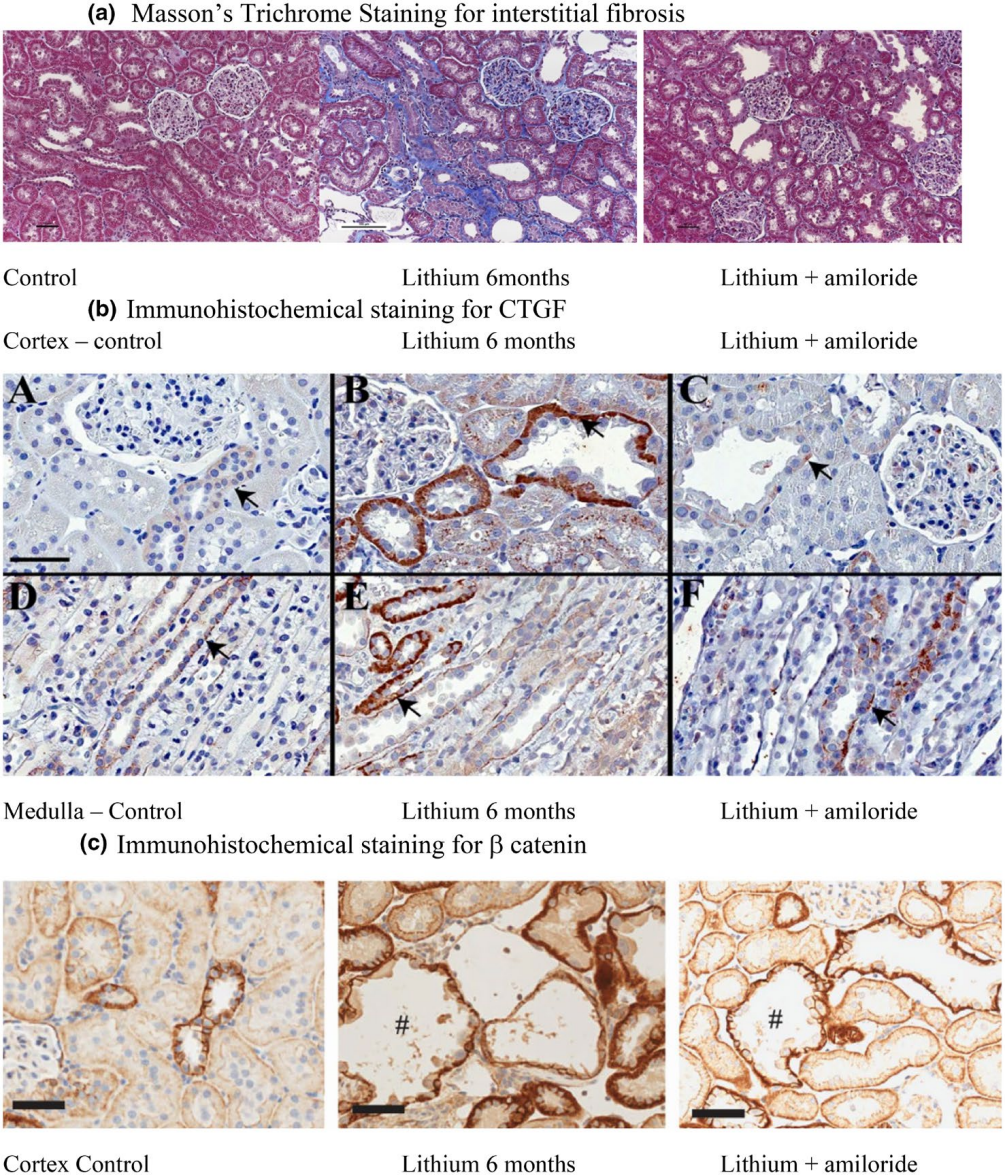
Rats were fed lithium carbonate (60 mmol/kg dry food) for 6 months or lithium carbonate (60 mmol/kg dry food) for 6 months with amiloride (0.2 mmol/L in drinking water) commenced after 4 weeks. (a) Masson's trichrome staining for interstitial fibrosis (collagen deposition staining blue) Magnification ×20. Scale bar 50 or 100 μm. (b) Immunohistochemical staining for CTGF in both cortical and medullary segments. Magnification × 200. Scale bar 50 μm. (c) Immunohistochemical staining for β catenin in cortical sections. Scale bar 50 μm. Magnification × 400. Modified from Walker et al. (2013), Kalita‐De Croft et al. (2018)), and used under the CC‐BY Creative Commons License from Mehta et al. (2022)).

Long‐term lithium exposure in a rat model results in a progressive increase in interstitial fibrosis with increased collagen deposition, increased interstitial macrophages, and alpha smooth muscle actin (αSMA) positive myofibroblasts (Walker et al., 2013). This was accompanied by increased TGFβ, CTGF with upregulation of the metalloproteinases consistent with excess extracellular matrix deposition (Figure 2) (Marti et al., 2016; Walker et al., 2013). Mehta and colleagues used RNA sequencing and microRNA expression analysis from kidney cortices obtained from rats exposed to lithium over 14 days, 28 days and 6 months to explore the intracellular pathways associated with progressive lithium‐induced interstitial fibrosis (Mehta et al., 2022). They demonstrated lithium induced overexpression of number of intracellular pathways including “cell cycle signalling”, “NF‐κB signaling”, “p53 signaling”, “Wnt signaling”, and “aldosterone up‐regulated genes”. Subsequent validation of candidate genes found inflammatory components induced by lithium including NF‐κB/p65^Ser536^ and activated pAKT^Ser473^, and increased p53 mediated regulatory function through increased p21 expression in damaged tubular epithelial cells (Mehta et al., 2022). They also identified that PDGFrβ expression was increased following lithium exposure, and co‐labelling of kidney cortical sections with αSMA and PDGFrβ showed sparse but distinct co‐localisation of PDGFrβ positive pericytes to αSMA labeled myofibroblasts in the kidney cortical interstitium around dilated tubules. This suggested that activated pericytes may be recruited and mediate, in part, the lithium‐induced interstitial fibrosis (Mehta et al., 2022).

**FIGURE 3:**
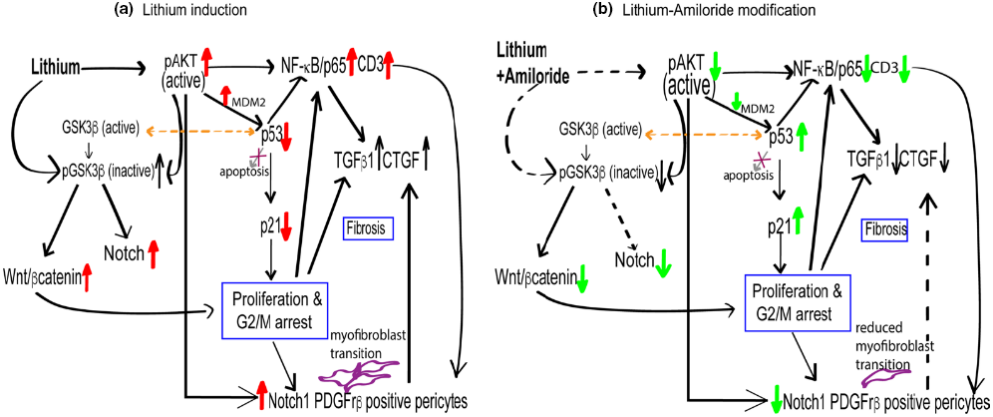
An overview of the intracellular pathways upregulated following lithium therapy (shown with red arrows) and those that are downregulated with the addition of amiloride (shown with green arrows). Used under the CC‐BY Creative Commons License from Mehta et al. (2022).

Therefore, these results suggest lithium exposure leads to increased Wnt/β‐catenin pathway activation, in part via lithium‐induced phosphorylation of GSK3β, which causes tubular epithelial cell G2/M growth arrest (Mehta et al., 2022). Lithium exposure also induces a progressive increase in pAKT (active), increased NF‐κB/p65 signaling, recruitment of infiltrating CD3 T cells, and decreased cellular p53 function (Mehta et al., 2022). The increased inflammation is associated with increased expression of the profibrotic cytokines TGFβ1 and CTGF (Bedford, Leader, et al., 2008; Hestbech et al., 1977) with subsequent fibrosis and inflammation (Marti et al., 2016; Mehta et al., 2022). Lithium‐induced tubular epithelial cell injury also promotes PDGFβ production, with recruitment and accumulation of PDGFrβ positive pericytes, which have features of myofibroblasts, further contributing to the development of interstitial fibrosis (Mehta et al., 2022) (Figure 3).

## 5 AMILORIDE DOWNREGULATES LITHIUM‐INDUCED INTERSTITIAL FIBROSIS

Our research group has previously demonstrated that long‐term (6 months) co‐treatment with amiloride in lithium‐exposed rats, initiated after 4 weeks of lithium exposure, significantly slowed the development of interstitial fibrosis with decreased expression of TGFβ and CTGF, reduced myofibroblast infiltration, and decreased extracellular matrix (Figure 2) (Mehta et al., 2022). Using RNA sequencing, in both short‐term (2–4 weeks) and long‐term (6 months) models, we identified that co‐treatment with amiloride attenuated the expression of activated Akt, p53 and its downstream effector p21, NF‐κB/p65, and the expression of CD3‐positive T cells. The protective effects of lithium‐amiloride co‐treatment—characterized by increased p53 and p21 stabilization, reduced Notch1 expression, and decreased NF‐κB/p65‐mediated inflammation—may be mediated through reduced levels of inactive pGSK3β with decreased expression of β catenin (Figure 2). This mechanism is consistent with previous findings showing that active GSK3β binds to and transactivates p53 (Watcharasit et al., 2003), while simultaneously inhibiting Notch signaling (Wang et al., 2019), as observed in the lithium‐amiloride treated samples (Figure 3) (Mehta et al., 2022).

## 6 SUMMARY

In summary, animal models of lithium‐induced chronic kidney interstitial fibrosis perhaps more accurately reflect the slowly progressive CKD as seen in humans, unlike other models such as 1 ¾ nephrectomies or unilateral ureteric obstruction, which produce a rapidly progressive interstitial fibrosis. The impact of lithium is multifaceted, affecting many intracellular cell signaling pathways, cell cycle regulation, cell proliferation, and subsequent interstitial fibrosis. Thus, models of lithium‐induced changes in cellular function have the potential to expand our knowledge of pathways of tubular cell injury and repair (Kishore & Ecelbarger, 2013). Amiloride appears to have multiple pleiotropic actions, independent of its competitive antagonism of ENaC in reducing the progressive interstitial fibrosis. One action revealed was a role for amiloride in sustaining p53 function. Further exploration of these interactions will lead to potential new therapeutic targets and drugs.
